# Branching out or bearing fruit: how MADS-box transcription factors orchestrate maize ovule development

**DOI:** 10.1093/plcell/koag296

**Published:** 2026-09-23

**Authors:** Ved Prakash

**Affiliations:** Assistant Features Editor, the Plant Cell, American Society of Plant Biologists; Department of Plant Pathology, College of Food, Agriculture, and Environmental Sciences, The Ohio State University, 1680 Madison Ave, Wooster, OH 44691, United States

New plant organs originate from meristems which contain a pool of pluripotent stem cells. Shoot apical meristem (SAM), root apical meristem (RAM) and floral meristem (FM) produce arial, underground, and floral tissues, respectively (Carles and Fletcher 2003; Perilli et al. 2012). In flowering plants, the formation of seed depends on timely transition of pluripotent FMs into specialized, determinate floral organs. The classical ABC(DE) model of floral development explains how varying combinations of transcription factors contribute to the identity of different parts of a flower (Bowman and Moyroud 2024). However, the point at which flowering development transitions from organogenesis to ovule formation, particularly in cereal crops, is not well understood.

In maize (*Zea mays*), the pistil is made up of 3 carpels. Two of the carpels grow out and elongate to give rise to silk (style) while the third carpel is shorter, contains a single fertile ovule, which after fertilization develops into a kernel (Klein et al. 2022). The flower development in maize follows the ABCDE model in which *ZmMADS8* and *ZmMADS14* transcription factors are required for carpel formation. Loss-of-function mutants fail to produce carpels and instead exhibit prolonged meristem activity and repeatedly produce indeterminate, branch-like structures.

Using these genetic mutants as tools to understand FM regulation in maize, **Yunfu Li and colleagues (**Li et al. 2026) applied single-cell RNA sequencing (scRNA-seq) to understand how ZmMADS8/14 regulate meristem termination and ovule development in maize. Histology and 5-ethynyl-2'-deoxyuridine (EdU) incorporation assays (to label cells actively proliferating) demonstrated that wild-type (WT) FM apex cells exit the cell cycle so that the embryo sac (gametophyte) could differentiate, while the *mads8/14* mutant remained in a highly proliferative state with smaller, densely packed cells. To further characterize these differences between WT and *mads8;14* FM, the authors employed scRNA-seq on over 28,000 single cells from FM tissues of the 2 genotypes. Cell clustering and pseudotime trajectory analyses revealed that mutant cells are significantly less differentiated than WT cells, suggesting that *ZmMADS8* and *ZmMADS14* mainly regulate floral organ cell specialization and differentiation.

By utilizing machine learning (XGBoost) and co-expression analysis, the authors identified *ZMM3*, another MADS-box transcription factor, as significantly upregulated in *mads8*;*14*. While *ZMM3* expression was restricted to young FM and nucellus in WT florets, its expression in *mads8;14* was ectopically upregulated across placenta and branch-like structures. Using chromatin immunoprecipitation (ChIP)-qPCR, the authors found that ZmMADS8 directly binds a CArG-box motif within the *ZMM3* promoter, confirming *ZMM3* as a direct downstream target repressed by ZmMADS8/14. Furthermore, by using comparative transcriptomics, the authors revealed that elevated levels of ZMM3 suppresses key floral organ identity genes and ovule/gametophyte regulators, while activating meristem maintenance genes. These findings establish that ZmMADS8/14 promotes floral organogenesis and ovule fate by negatively regulating *ZMM3* levels and keeping its expression spatially confined to the nucellus (see Fig. 1).

**Figure 1:**
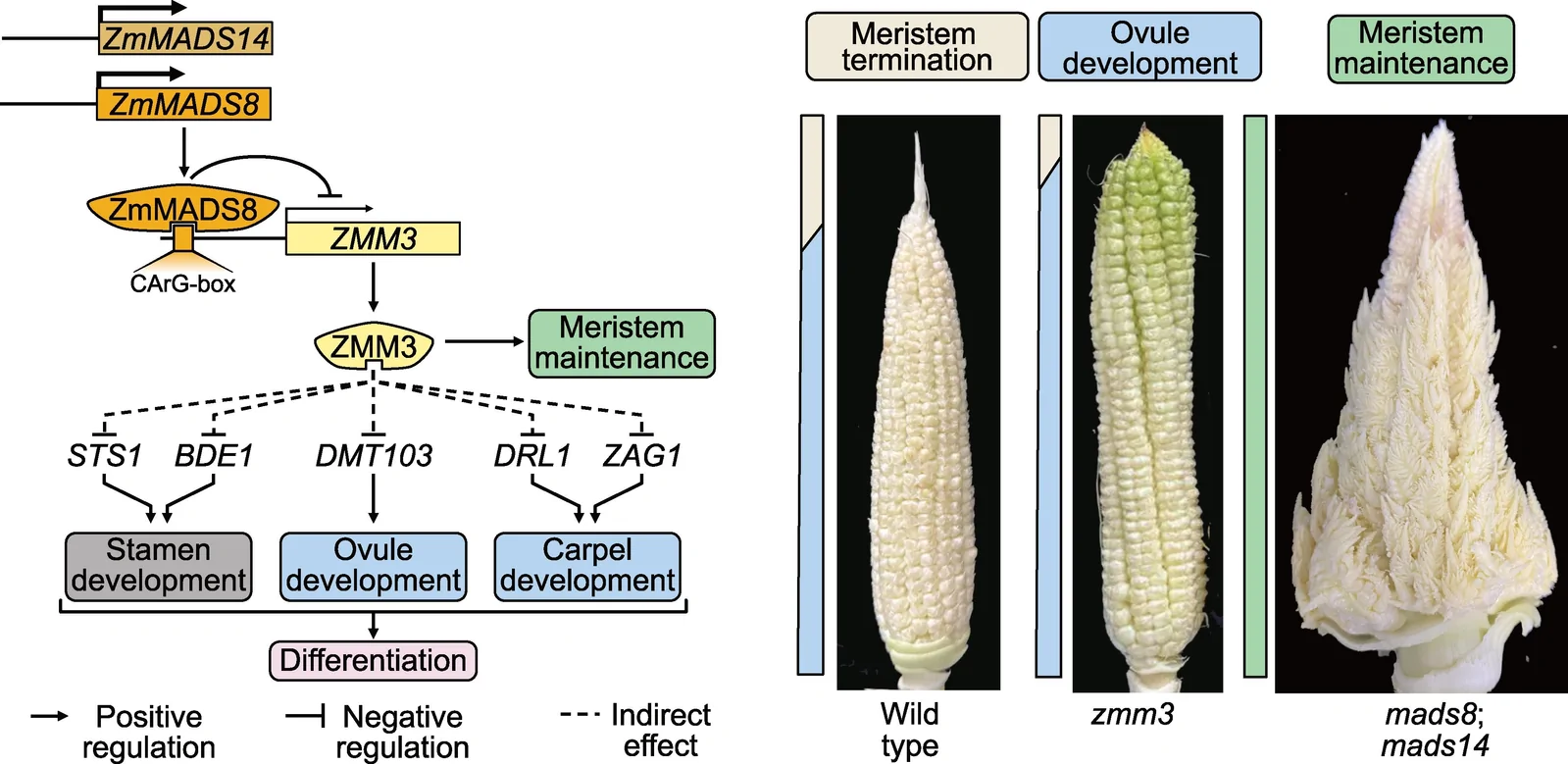
A proposed model depicting the role of *ZmMADS8/14* in regulating maize floral organ cell specialization and differentiation. *ZmMADS8/14* activate organ identity genes (*STS1, BDE1*, *ZAG1* and *DRL1*/*IFA1*) and nucellus factor (*DMT103*), while repressing meristem maintenance factor *ZMM3.* Adapted from Li et al (2026), Fig. 8.

Knocking out *ZMM3* in the *mads8;14* background partially rescued the mutant phenotype. Interestingly, knockout of *ZMM3* alone in WT backgrounds increased the number of fertile florets per row, leading to the formation of longer ears and increased number of kernels. After crossing with the parent inbred lines of commercial Chinese hybrids, the *zmm3* knockout allele promoted fertility by increased ear length and kernel number.

By combining single-cell transcriptomics, histological profiling, and functional genetics, the authors revealed key factors influencing maize ovule initiation. This study not only characterizes the regulation of FM termination but also demonstrates how exploring developmental regulatory networks can identify valuable genetic targets for engineering high-yielding cereal crops.

## Recent related articles in *The Plant Cell*

- Wu et al. (2025) demonstrated mechanism by which blue light is perceived and flowering is regulated in maize. The findings offer a promising strategy for optimizing crop performance in diverse environments.
- Creach et al. (2026) used multi-omics data to predict maize phenotypes. The authors integrated transcriptomics and phenomics data with genetics and demonstrated that such integration enhanced trait prediction across environments and provided crucial information into the genetic and regulatory architecture of agriculturally valuable traits in maize.
- Zhao et al. (2026) characterized an EMS-induced *additive delayed-flowering* mutant, *edf1,* in maize which possess a splice-donor mutation in *ALTERED PHLOEM DEVELOPMENT (APL)*. They reported that *APL* is a valuable target for base editing to engineer flowering time.

## Data Availability

No new data were generated or analysed in support of this research.
